# Relative dose intensity delivered to patients with early breast cancer: Canadian experience

**DOI:** 10.3747/co.v16i6.311

**Published:** 2009-12

**Authors:** S. Raza, S. Welch, J. Younus

**Keywords:** Early breast cancer, adjuvant chemotherapy, relative dose intensity

## Abstract

Adjuvant chemotherapy for early breast cancer improves disease-free and overall survival in pre- and postmenopausal women. The importance of maintaining relative dose intensity (rdi) is well-known; however, little information is available from routine clinical practice regarding how well dose intensity is maintained with modern chemotherapy regimens.

In a retrospective review of patients undergoing chemotherapy for early breast cancer at a single institution in Canada from January 2006 to November 2007, a total of 263 patients received one of the following regimens:

- ac-t [doxorubicin (Adriamycin: Pharmacia, Kalamazoo, MI, U.S.A.)–cyclophosphamide, paclitaxel (Taxol: Bristol–Myers Squibb, Princeton, NJ, U.S.A.)]
- fec-100 (5-fluorouracil–epirubicin–cyclophosphamide)
- fec-d (5-fluorouracil–epirubicin–cyclophosphamide, docetaxel)

Overall, only 14.4% of patients had a rdi less than 85%. Dose delay or reduction (or both) occurred in 46%, 37%, and 20% of patients receiving fec-100, ac-t, and fec-d respectively. Optimal rdi was delivered to 96%, 95%, and 70.7% of patients for ac-t, fec-d and fec-100 regimens respectively. Patients over 65 years of age accounted for 14% of the total cohort and were more likely to receive a suboptimal rdi than were patients younger than 65 years of age (35% vs. 6.6%).

Optimal chemotherapy rdi (>85%) for early breast cancer can be achieved at an academic cancer centre. This goal is less often accomplished in elderly patients, and thus a proactive approach is required for managing toxicity in that population.

## 1. INTRODUCTION

Breast cancer is the most common form of cancer affecting women in Canada, with more than 22,200 new cases being diagnosed and 5300 deaths occurring annually 1. Early breast cancer (ebc) is considered to be a potentially curable disease. Surgery remains the definitive treatment for ebc. Adjuvant systemic therapy, such as chemotherapy or hormonal treatment, is administered depending upon stage, grade, and other tumour characteristics.

In premenopausal women, adjuvant chemotherapy reduces the risk of disease relapse by 37% and of death by 30%. For women aged 50 to 69 years, the risk reductions are 19% (relapse) and 12% (death). The conferred absolute gain in survival appears to depend on the magnitude of risk of recurrence at presentation 2.

To achieve the full benefit of chemotherapy in potentially curable ebc, maintaining dose intensity is very important. In chemotherapy regimens, there is good evidence for a threshold of delivered relative dose intensity (rdi), below which the clinical benefits may become compromised 3. Many clinicians and quality assurance programs have adopted the rdi criterion of Bonadonna *et al.* in adjuvant chemotherapy for ebc 3.

The benefit of maintaining dose intensity is not limited to breast cancer. Trials in non-Hodgkin lymphoma (nhl) have demonstrated similar improvements in disease-free survival and overall survival if dose intensity is maintained above a certain level 4,5.

Despite the fact that maintaining rdi is important to achieve improved outcome, many patients in adjuvant settings are treated with a lower dose intensity of chemotherapy. Lyman *et al.* reported a survey of more than 20,000 women with ebc, which showed that 55% of women received less than 85% of the rdi 6. A similar trend of dose reduction was reported in more than 4500 patients with aggressive nhl. In that survey, dose reductions of 15% or more occurred in 40% of patients, and treatment delays up to 7 days occurred in 24% of patients 7. Most of the patients in the ebc study reported by Lyman *et al.* 6 received cmf [cyclophosphamide, methotrexate, 5-fluorouracil (5fu)], caf (cyclophosphamide, doxorubicin, 5fu), or ac [doxorubicin (Adriamycin: Pharmacia, Kalamazoo, MI, U.S.A.), cyclophosphamide] chemotherapy regimens, which are now less frequently used for adjuvant treatment of ebc.

Considering the importance of maintaining dose intensity in the adjuvant setting 8, we conducted a retrospective analysis in patients with ebc treated at a Canadian centre with adjuvant chemotherapy consisting of fec-100 (5fu–epirubicin–cyclophosphamide), ac-t [doxorubicin (Adriamycin)–cyclophosphamide, paclitaxel (Taxol: Bristol–Myers Squibb, Princeton, NJ, U.S.A.)], or fec-d (5fu–epirubicin–cyclophosphamide, docetaxel). We also analyzed the incidence of febrile neutropenia in this patient population. Here, we report our experience with delivered rdi in these patients receiving the most recent generation of adjuvant chemotherapy regimens.

## 2. PATIENTS AND METHODS

After obtaining local ethics board permission to conduct the study, data were collected retrospectively in a chart review of 275 ebc patients treated from January 2006 to November 2007 at the London Regional Cancer Program, London, Ontario, Canada. Chart data was extracted only for those patients (Table I) who were treated with one of the following regimens: ac-t (4 cycles of doxorubicin–cyclophosphamide, followed by 4 cycles of paclitaxel, all given once every 3 weeks), fec-100 (6 cycles of 5fu–epirubicin–cyclophosphamide, once every 3 weeks), or fec-d (3 cycles of fec-100 followed by 3 cycles of docetaxel, all given once every 3 weeks).

Data collection included pretreatment demographics and clinical characteristics, particularly age at diagnosis, chemotherapy regimen, and planned dose and schedule. All episodes of febrile neutropenia were also recorded for each patient across all cycles.

The primary objective was to calculate the delivered rdi, defined as the proportion actually received of the reference standard dose intensity for each regimen. The numbers of patients receiving less or more than 85% of the rdi were recorded separately. Incidences of chemotherapy dose delays of more than 7 days or dose reductions of more than 15% were recorded for all chemotherapy cycles.

Summative dose intensity (sdi) is a concept applied to clinical trials in which the relationships of dose intensity with effects are studied. The sdi is defined as the sum of the contributions of individual drug dose intensities in a combination drug regimen, and here, it is calculated based on the work of Hryniuk *et al.* 12 Hryniuk *et al.* showed a positive relationship between higher dose intensities and better outcomes in patients with ebc, and those data have served as the groundwork for many trials starting in the late 1980s.

The sdi of each regimen is calculated in four steps:

- First, a unit dose intensity (udi—the dose of the drug required to produce 30% complete responses, plus partial responses, when used as a first-line single-agent chemotherapy in metastatic breast cancer) is calculated for each drug in the combination regimen. The udis for 5fu, epirubicin, and cyclophosphamide are 650, 25, and 700 respectively.
- In the second step, the dose intensity of each drug in a combination is expressed as a fraction of it own udi. The individual dose in milligrams per square metre body surface area is converted to a per-week fraction. For example, in the fec-100 regimen, cyclophosphamide and 5fu at 500 mg/m^2^ every 3 weeks becomes 167/week, and epirubicin at 100 mg/m^2^ every 3 weeks becomes 33.33/week.
- In the third step, the calculated weekly standard dose from step 2 is divided by the udi from step 1 to yield the sdi of the individual drug (Table II shows the sdis of the studied regimens). For example, for 5fu, 167/650 produces an sdi of 0.26. The corresponding results for epirubicin and cyclophosphamide are 1.33 and 0.23 respectively.
- In the last step, the sdis of the regimen are summed. For fec-100, the final sdi is therefore 1.82.

If a patient experiences a dose reduction of 25% in 6 cycles of fec-100, the drop for the total dose of 5fu would be to 2250 mg/m^2^ from 3000 mg/m^2^ over 18 weeks. The corresponding dose intensity would be calculated at 125/week to yield a final sdi of 0.178. In this case scenario, the sdis for epirubicin and cyclophosphamide would be 1 and 0.192, thereby delivering a 1.37 sdi for the regimen. Thus, with this reduction in the dose, the delivered rdi is 1.37/1.82 (about 75%). In randomized trials in which dose intensity is tested, response rates and survival were linearly associated with the sdi in each treatment arm 6.

## 3. RESULTS

Of the 263 evaluable patients, 98 received fec-d; 100, ac-t; and 65, fec-100. Overall, 14.4% of the patients experienced a dose delay of 1 week or more, and 22% experienced a dose reduction of any degree (Table I). About 33% of all patients experienced either dose reduction or dose delay, but overall only 14.4% received less than 85% of the rdi.

**FIGURE 1:**
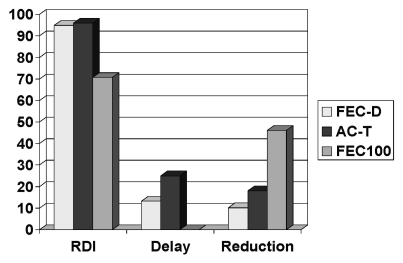
Dose delay, dose reduction, and relative dose intensity (rdi) for the chemotherapy regimens. fec-d = 5-fluorouracil(5fu) epirubicin cyclophosphamide, docetaxel; act-t = doxorubicin (Adriamycin: Pharmacia, Kalamazoo, MI, U.S.A.) cyclophosph-amide, paclitaxel (Taxol: Bristol Myers Squibb, Princeton, NJ, U.S.A.); fec-100 = 5fu, epirubicin, cyclophosphamide.

Dose delays were noted in 25%, 13%, and 0% of patients receiving ac-t, fec-d, and fec-100 respectively, and dose reductions were noted in 18%, 10.2%, and 46% respectively. Dose delay or reduction was found in 46% of patients treated with fec-100, in 37% treated with ac-t, and in 20% treated with fec-d. The delivered rdi was above 85% in 96%, 95%, and 70.7% of the ac-t, fec-d, and fec-100 regimens (Figure 1).

In our study population, 14% patients were 65 years of age or older. Of the patients treated with fec-100, 33.8% were over 65 years of age as compared with 8.2% of patients treated with fec-d and 7% treated with ac-t.

**FIGURE 2:**
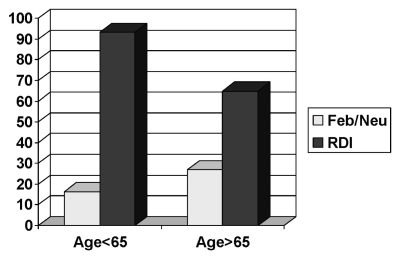
Febrile neutropenia (Feb/Neu) and relative dose intensity (rdi) in the study population, by patient age.

**FIGURE 3:**
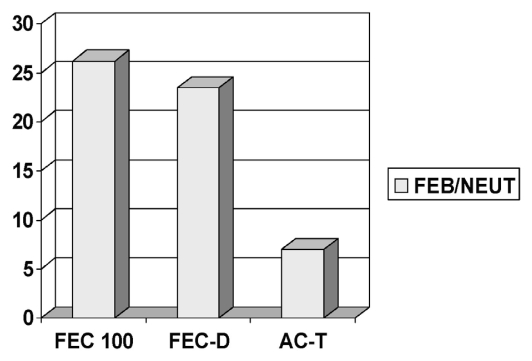
Rate of febrile neutropenia (feb/neut) by chemotherapy regimen. fec100 = 5-fluorouracil (5fu) epirubicin cyclophosphamide; fec-d = 5fu epirubicin cyclophosphamide, docetaxel; ac-t = doxorubicin (Adriamycin: Pharmacia, Kalamazoo, MI, U.S.A.) cyclophosphamide, paclitaxel (Taxol: Bristol Myers Squibb, Princeton, NJ, U.S.A.).

The delivered rdi was less than 85% in approximately 35% of patients 65 years of age or older, as compared with 6.6% of patients younger than 65 years of age. Overall, for the 65-or-older age group, the incidence of febrile neutropenia was 27% as compared with 16.3% for patients under 65 years of age (Figure 2). The overall incidence of febrile neutropenia was 17.8%, being observed at rates of 26.1%, 23.4%, and 7% in the fec-100, fec-d, and ac-t regimens respectively (Figure 3). Most of the febrile neutropenia episodes in patients treated with the fec-d regimen (19 of 23) coincided with the first cycle of docetaxel.

## 4. DISCUSSION AND CONCLUSIONS

### 4.1 Delivered RDI

We analyzed data for patients receiving three frequently used adjuvant chemotherapy regimens for ebc, utilizing appropriate methods to calculate the delivered rdis. Considering the importance of rdi, we hope that our data will help other physicians to calculate rdi for their patients. Data from previously published trials demonstrate that ebc patients treated with adjuvant chemotherapy are at considerable risk of reduced delivered rdi 6,7. However, our data analysis has been encouraging: It showed that the rdi received by most of our patients exceeded 85%.

This result is likely attributable to multiple factors. Our medical oncology group adheres to breast cancer treatment guidelines when evaluating systemic therapy plans for patients with ebc. There is general awareness among medical oncologists about the importance of the relationship between dose intensity and outcome in ebc. Several previous trials have provided data showing improved outcomes with maintenance of rdi during adjuvant chemotherapy for ebc 13,14. A 20% dose reduction may compromise cure by 50%, and patients receiving less than 65% dose intensity are expected to have a survival similar to that of an untreated control group 15. Randomized clinical trials have shown that granulocyte colony–stimulating factor (g-csf) can reduce the risk of complications associated with chemotherapy and also facilitate the delivery of full-dose rdi 16. The accessibility and more frequent use of growth factors could be another reason behind our results. For the patients reviewed here, the use of g-csf was limited to patients with documented febrile neutropenia, and none of the patients was treated with g-csf as primary prophylaxis. Earlier surveys showing a lower delivered rdi in ebc patients focussed on community medical oncology practices. Our centre, being an academic cancer centre, may certainly have had a different approach to managing patients from the perspective both of the physicians and the nursing staff and of resources, accounting for a different outcome.

### 4.2 Occurrence and Management of Febrile Neutropenia

Generally, elderly women are considered to be more prone to chemotherapy-related side effects, including febrile neutropenia, which may have translated to a reduced rdi for this group of women within the study sample. Among fec-100 treated patients, the incidence of febrile neutropenia was high, and more frequently, these patients received a lesser rdi. However, a relatively smaller number of patients were treated with the fec-100 regimen. These findings correlate well with the fact that 34% of patients treated with the fec-100 regimen were more than 65 years of age (compared with 16% and 7% of the patients treated with fec-d and ac-t). In addition, the fec-100 recipients appeared to have more comorbid illnesses than did patients receiving other adjuvant combination chemotherapy.

The fec-d regimen is an effective, relatively new, but quite frequently-used combination for ebc in the adjuvant setting. The reported incidence of febrile neutropenia with fec-d in the pacs 01 trial (Sequential Adjuvant Epirubicin-Based and Docetaxel Chemotherapy for Node-Positive Breast Cancer Patients) was around 11% 10; however, our experience shows a higher risk of febrile neutropenia (23%). Interestingly, most of the febrile neutropenia incidences were reported with the first dose of docetaxel (cycle 4), consistent with the observations in the pacs 01 trial. Other Canadian cancer centres have experienced similar trends in the incidence of febrile neutropenia with the fec-d regimen (Ottawa and Sudbury cancer centres. Personal communication). With such a high rate of febrile neutropenia, we should consider giving g-csf as primary prophylaxis with fec-d, possibly starting from cycle 1, but at least for the last 3 cycles.

### 4.3 Study Limitations

The present study is limited by its retrospective design and small sample size. However, we feel that it still represents a reasonable cross-sectional view of the management of ebc in a Canadian academic cancer centre. The three regimens analyzed were in use at different times. The ac-t combination was used between 2001 and 2004, and many patients included in the review were part of the National Cancer Institute of Canada Clinical Trials Group ma.21 trial. Thus a potential selection bias related to those patients may be present. The fec-100 regimen came into use around 2005 and was initially believed to be carrying relatively fewer side effects. That belief may have contributed more elderly patients to that group. The fec-d regimen is the newest and has been particularly used in lymph-node-positive patients. Thus, almost a decade has gone into the selection of these patients, and changes in physician practices, guidelines, and other factors may potentially have injected some biases into the outcomes reported.

### 4.4 Summary

We hope that these data can help physicians to become more aware of the importance of maintaining optimal dose intensities for adjuvant chemotherapy treatment in ebc. We believe that improvements in the overall management of such patients will lead to doses in the frequently used chemotherapy regimens being maintained close to the optimal rdi. Because elderly patients derive benefits from the use of adjuvant chemotherapy similar to those seen in their younger peers, particular attention should be paid to the need to improve delivered rdi in the elderly patient population. A proactive role to address chemotherapy-related side effects and the use of g-csf may help to achieve that goal.

## Figures and Tables

**TABLE I:** Characteristics of the treatment received by the study patients

Treatment characteristic	Overall (*N =* 263)	Patients Age<65 (86%)	Age ≥65 (14%)
ac/t	*n=*100	93%	7%
fec/d	*n=*98	91.8%	8.2%
fec 100	*n=*65	66.1%	33.9%
rdi > 85%	89.9%	93.3%	64.8%
Dose delay > 7 days	14.4%	15%	10.8%
Dose reduction	22%	16.8%	54%

rdi = relative dose intensity.

**TABLE II:** Chemotherapy regimens: reference standard dose, schedule, and summative dose intensity (sdi)

	ac/t^9^ (*n*=100)	fec/d^10^ (*n*=100)	fec 100 11 (*n*=75)
Cycle length (days)	21	21	21
Doxorubicin [a (mg/m^2^)]	60		
Cyclophosphamide [c (mg/m^2^)]	600	500	500
Paclitaxel [t (mg/m^2^)]	175		
Fluorouracil [f (mg/m^2^)]		500	500
Docetaxel [d (mg/m^2^)]		100	
Cycles given (*n*)	ac×4, t×4	fec×3, d×4	fec×6
sdi	3	3.8	1.8

## References

[1] Canadian Cancer Society and the National Cancer Institute of Canada Canadian Cancer Statistics 2006 Toronto Canadian Cancer Society 2006

[2] Early Breast Cancer Trialists’ Collaborative Group (ebctcg) Effects of chemotherapy and hormonal therapy for early breast cancer on recurrence and 15-year survival: an overview of the randomized trials Lancet 2005 365 1687 717 15894097 10.1016/S0140-6736(05)66544-0

[3] Bonadonna G Valagussa P Moliterni A Zambetti M Brambilla C Adjuvant cyclophosphamide, methotrexate, and fluorouracil in node-positive breast cancer: the results of 20 years of follow-up N Engl J Med 1995 332 901 6 7877646 10.1056/NEJM199504063321401

[4] Kwak LW Halpern J Olshen RA Horning SJ Prognostic significance of actual dose intensity in diffuse large-cell lymphoma: results of a tree-structured survival analysis J Clin Oncol 1990 8 963 77 2348230 10.1200/JCO.1990.8.6.963

[5] Lepage E Gisselbrecht C Haioun C Prognostic significance of received relative dose intensity in non-Hodgkin’s lymphoma patients: application to lnh-87 protocol. The gela (Groupe d’Etude des Lymphomes de l’Adulte) Ann Oncol 1993 4 651 6 7694634 10.1093/oxfordjournals.annonc.a058619

[6] Lyman GH Dale DC Crawford J Incidence and predictors of low dose-intensity in adjuvant breast cancer chemotherapy: a nationwide study of community practices J Clin Oncol 2003 21 4524 31 14673039 10.1200/JCO.2003.05.002

[7] Lyman GH Dale DC Friedberg J Crawford J Fisher RI Incidence and predictors of low chemotherapy dose-intensity in aggressive non-Hodgkin’s lymphoma: a nationwide study J Clin Oncol 2004 22 4302 11 15381684 10.1200/JCO.2004.03.213

[8] Wood WC Budman DR Korzun AH Dose and dose intensity of adjuvant chemotherapy for stage ii, node-positive breast carcinoma N Engl J Med 1994 330 1253 9 8080512 10.1056/NEJM199405053301801

[9] Mamounas EP Bryant J Lembersky B Paclitaxel after doxorubicin plus cyclophosphamide as adjuvant chemotherapy for node-positive breast cancer: results from nsabp B-28 J Clin Oncol 2005 23 3686 96 15897552 10.1200/JCO.2005.10.517

[10] Roché H Fumoleau P Spielmann M Sequential adjuvant epirubicin-based and docetaxel chemotherapy for node-positive breast cancer patients: the fnclcc pacs 01 Trial J Clin Oncol 2006 24 5664 71 17116941 10.1200/JCO.2006.07.3916

[11] Bonneterre J Roché H Kerbrat P Epirubicin increases long-term survival in adjuvant chemotherapy of patients with poor-prognosis, node-positive, early breast cancer: 10-year follow-up results of the French Adjuvant Study Group 05 randomized trial J Clin Oncol 2005 23 2686 93 15837983 10.1200/JCO.2005.05.059

[12] Hryniuk W Frei E 3rd Wright FA A single scale of comparing dose intensity of all chemotherapy regimens in breast cancer: summation dose intensity J Clin Oncol 1998 16 3137 47 9738586 10.1200/JCO.1998.16.9.3137

[13] Mayers C Panzarella T Tannock IF Analysis of prognostic effects of inclusion in a clinical trial and of myelosuppression on survival after adjuvant chemotherapy for breast carcinoma Cancer 2001 91 2246 57 11413512

[14] Shayne M Crawford J Dale DC Culakova E Lyman GH Predictors of reduced dose intensity in patients with early stage breast cancer receiving adjuvant chemotherapy Breast Cancer Res Treat 2006 100 255 62 16705366 10.1007/s10549-006-9254-4

[15] Skipper HE Kinetics of mammary tumor cell growth and implications for therapy Cancer 1971 28 1479 99 5127796 10.1002/1097-0142(197112)28:6<1479::aid-cncr2820280622>3.0.co;2-m

[16] Smith TJ Khatcheressian J Lyman GH 2006 Update of recommendations for the use of white blood cell growth factors: an evidence-based clinical practice guideline J Clin Oncol 2006 24 3187 205 16682719 10.1200/JCO.2006.06.4451

